# Multimodal Imaging Characteristics of a Large Retinal Capillary Macroaneurysm in an Eye With Severe Diabetic Macular Edema: A Case Presentation and Literature Review

**Published:** 2020-01-01

**Authors:** Omer Karti, Sefik Can Ipek, Ali Osman Saatci

**Affiliations:** 1 Department of Ophthalmology, İzmir Democracy University, İzmir, Turkey.; 2 Department of Ophthalmology, Ağrı State Hospital, Ağrı, Turkey.; 3 Department of Ophthalmology, Dokuz Eylul University Medical Faculty, İzmir, Turkey.

**Keywords:** Diabetic Retinopathy, Fluorescein Angiography, Indocyanine Green Angiography, Macroaneurysms, Microaneurysm, Optical Coherence Tomography

## Abstract

Though microaneurysms are the hallmark of diabetic retinopathy (DR), large aneurismal changes termed as ''macroaneurysms'' (MAs) may also occur in the course of chronic diabetic macular edema. MAs are usually accompanied by intraretinal hard exudates, fluid accumulation and retinal hemorrhages. Detection of MAs is clinically important as it implies that macular edema is usually chronic and therefore can be resistant to intravitreal anti-vascular endothelial growth factor injections. Multimodal imaging consisting of fluorescein angiography (FA), indocyanine green angiography (ICGA), optical coherence tomography (OCT) or OCT-angiography (OCTA) can be performed to detect and understand the nature of MA and thereby select proper treatment modality. Herein, we report multimodal imaging features of a 64-year-old woman with insulin-dependent diabetes mellitus presented with treatment naïve severe macular edema and a macroaneurysm at the right temporal macula. In conclusion, FA, ICGA and OCT seem to be far superior to OCTA to detect these lesions due to probable slow flow inside the MA.

## INTRODUCTION

Microaneurysms, hard exudates, dot or blot hemorrhages, macular edema, capillary occlusion, cotton-wool spots and neovascularization are among the clinical features of diabetic retinopathy (DR) [1]. Though the presence of microaneurysms is the clinical hallmark of DR, a few of the recent studies have reported that macroaneurysms (MAs) may also occur during the course of DR [2-4]. Retinal findings can be marked when MA is ruptured as the consequence of focal destruction of inner blood-retinal barrier. MAs may not be recognized on fundus examination or fluorescein angiography (FA). Therefore, other imaging methods such as indocyanine green angiography (ICGA) optical coherence tomography (OCT) and OCT-angiography (OCTA) may be required to detect MAs and understand the underlying pathophysiologic mechanisms [2, 4-6]. 

Herein, we demonstrated multimodal imaging (FA, ICGA, OCT, OCTA) features of a retinal MA in a patient with insulin-dependent diabetes mellitus (IDDM) and severe treatment naïve diabetic macular edema (DME).

## CASE REPORT

**Figure 1:**
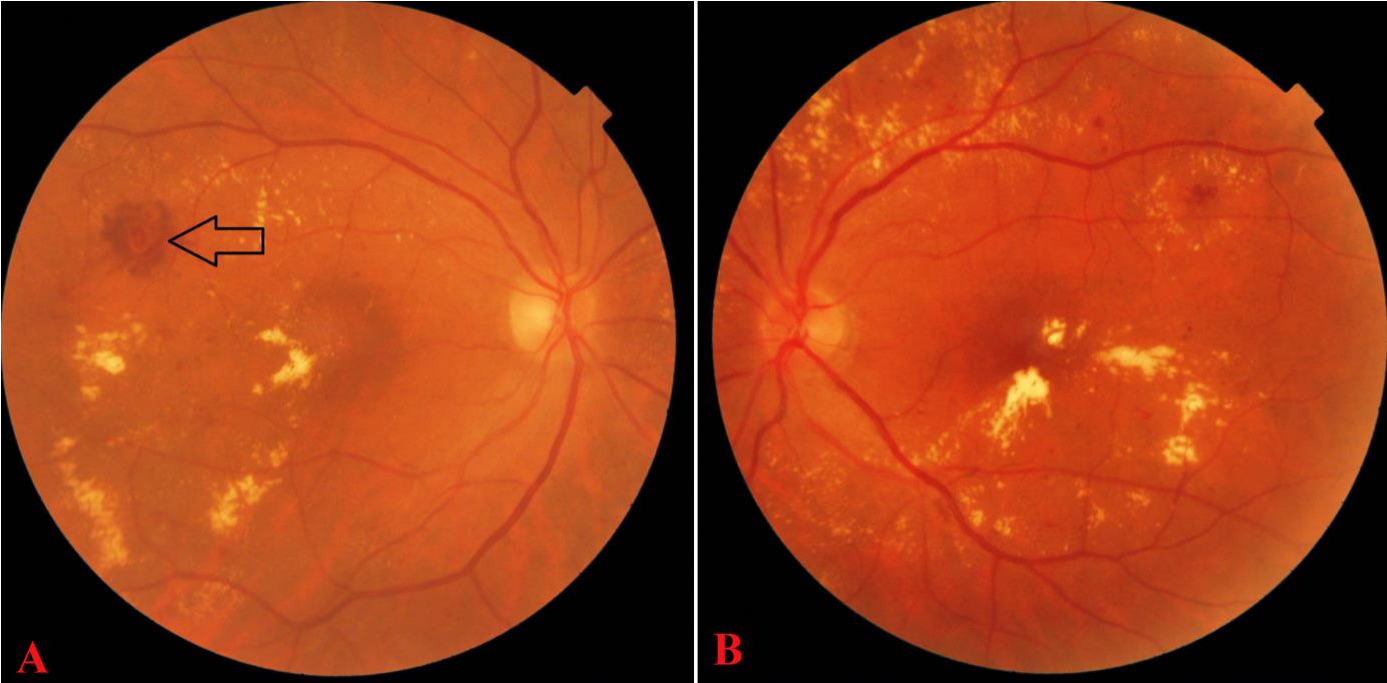
Fundus photographs of the right (A) and left eyes (B) showing venous dilatation blot hemorrhages, multiple microaneurysms and hard exudates. Fundus photograph of the right eye (A) illustrating macroaneurysm at the superior temporal area of the fovea (black arrow).

A 64-year-old female with IDDM presented with a gradual visual decline in both eyes (OU) and examined in October 2019 at Department of Ophthalmology (Dokuz Eylul University, İzmir, Turkey). Patient consent was obtained to write this case report. This study was performed in compliance with the principles of the Declaration of Helsinki. She had IDDM for 18 years with a recent HbA1c level of 6.2%. On admission, her best-corrected visual acuity with Snellen chart was 0.4 in right eye (OD) and 0.5 in left eye (OS). Slit-lamp examination and intraocular pressure had normal findings in OU. A detailed fundus examination revealed multiple microaneurysms, blot hemorrhages and circinate exudates at the macula bilaterally. In addition, a MA was detected at the superior temporal area of the fovea in OD (Fig 1). 

**Figure 2:**
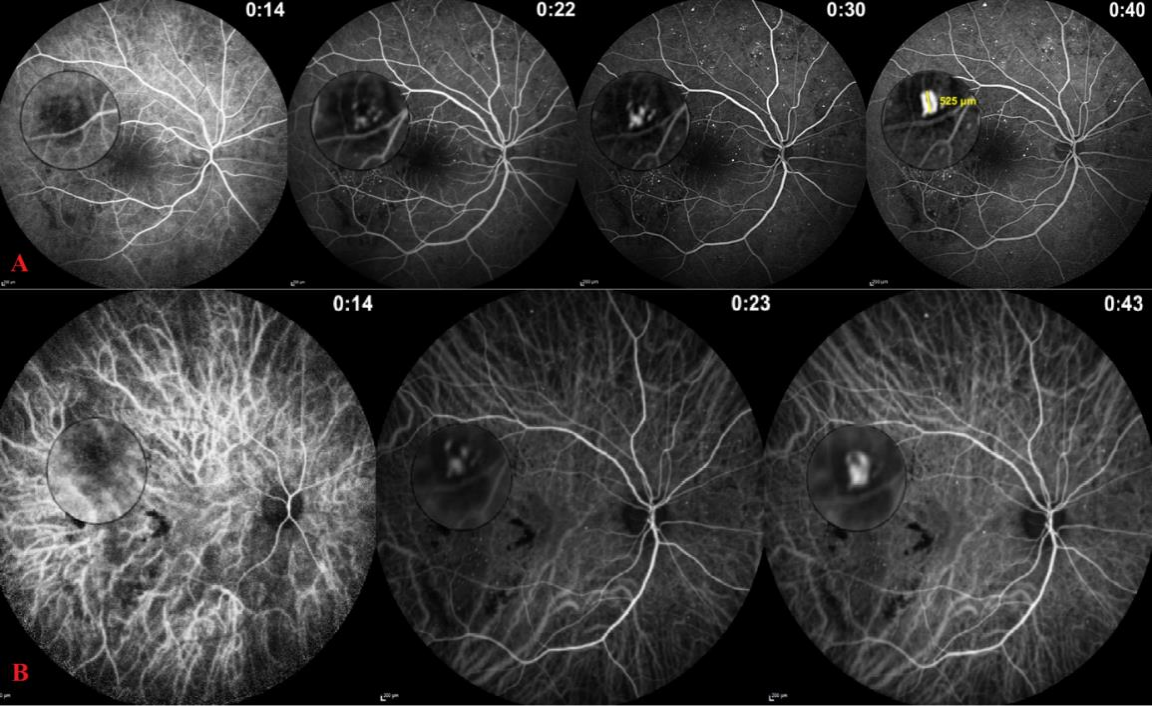
Angiographic images of the right eye illustrating macroaneurysm lesion and its progressive filling in magnified frames. Fluorescein (A) and indocyanine green angiogram (B) showing hypofluorescence lesion in early phase and slow dye filling in late phase.

FA showed multiple hyperfluorescent lesions in the early phase corresponding to microaneurysms in OU. In addition, a slow filling of large diameter hyperfluorescent lesion (A vertical diameter of about 525 micrometers (μm) with no leakage was observed at the superior temporal area of the fovea in OD (Fig 2A). Full lesion was noted to fill with dye at the 40th second of angiogram. Similarly, ICGA confirmed the presence of round shape hyperfluorescent lesion and full lesion could be filled at the 43th second of angiogram (Fig 2B). 

**Figure 3:**
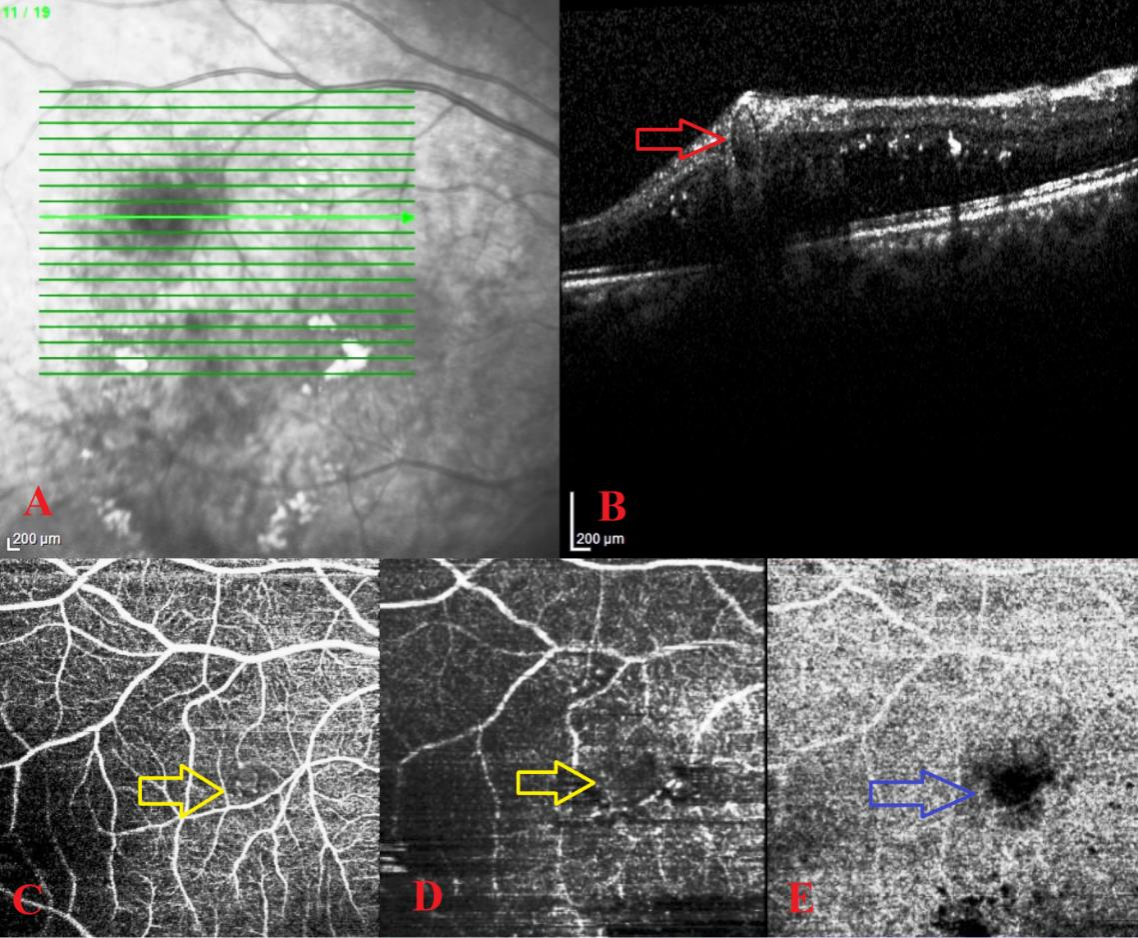
Optical Coherence Tomography (OCT) B scan (A, B) and OCT-Angiography (OCTA) scans (C-E) of the right eye obtained from Triton™ DRI swept‐source optical coherent tomography (SS‐OCT) instrument. OCT scan (B) passing through the macroaneurysm indicating an oval lesion (red arrow), with a hyperreflective wall, containing partially hyperreflective lumen, and back-shadowing, surrounded by intraretinal fluid. OCTA showing round-demarcated faded flow area at the level of superficial (C) and deep (D) capillary plexus (yellow arrows). OCTA image at the level of choriocapillaris (E) illustrating hypointense flow area corresponding to MA-related back-shadowing (blue arrow).

OCT scan of the right eye revealed an oval lesion corresponding to aneurysm. This lesion had a hyperreflective wall, containing partially hyperreflective lumen and back shadowing. Also, there was a thickened retina surrounding the MA, hyperreflective dots and serous subretinal fluid suggestive of inflammation **(**Fig 3A and B**)**. OCTA showed a round-demarcated faded flow area at the level of superficial and deep capillary plexus **(**Fig 3C and D**)**. Hypointense flow area corresponding to MA-related back-shadowing was noted at the choriocapillaris slab **(**Fig 3E**). **

Regarding these findings, severe non-proliferative DR, DME and DR-related MA were suggested. Although a combined treatment of direct focal argon laser photocoagulation onto the MA together with an intravitreal dexamethasone implant was recommended, but she denied any treatment due to reimbursement issues of dexamethasone implant. She was lost to follow-up.

## DISCUSSION

In this case presentation, comprehensive multimodal imaging features of a large retinal capillary MA were presented in a patient with IDDM and severe DME. 

DR-related MA was first described by Bourhis et al. [2] Because the term ''macroaneurysms'' could cause confusion with an arterial MAs [7-11], they recommended the use of ''telangiectatic capillaries''. Its prevalence and incidence remain still unknown in DR. They can develop from vessels including capillaries, arteries, veins or collaterals as a result of focal weakening of the vessel wall and endothelial cell proliferation [2]. Because of the severely defective site of the blood-retinal barrier, accumulation of large materials such as lipoproteins is prominent. MAs are more likely to be seen in areas close to the hard exudates [2, 4, 5]. In a series conducted by Castro Farías et al. [4], a significant correlation was found between the presence of MAs and severity of hard exudates. Similarly, Paques et al. [5] reported that the capillary MAs are often located at the center of circinate hard exudates. In the present case, the MA was closely associated with the circinate hard exudates as clearly depicted on the right color fundus picture and this clinical appearance is in accordance with aforementioned investigations.

MAs have diameters larger than 150 μm and localized very close to microaneurysms and share similar OCT and ICGA features [4, 5]. It is therefore suggested that MAs are originated from the microaneurysms [4, 12]. Although no clear-cut criteria have been identified to distinguish MAs and microaneurysms other than their size, some differentiating angiographic characteristics have been described. Microaneurysms show a bright contrast in the earlier phase, whereas no change in contrast size or brightness is observed in the later phase. By contrast, MAs often fill later than microaneurysms on the course of angiogram. Angiographic studies revealed a delayed or prolonged complete ICG filling. Also, fluorescence in MAs often persists longer in the ICGA examinations [2, 4, 5]. Similarly, our patient's angiogram showed early hypofluorescence of the MA with a relatively delayed complete dye filling.

Progressive staining of intraluminal material (proteins and/or lipids) is suggested to be the cause of delayed or prolonged dye filling in ICGA. Histological studies have also shown that intraluminal materials such as lipids and/or stagnant blood and even thrombosis have a high affinity for staining with ICG [3]. ICG that may stain intraluminal fibrinogen due to amphiphilic properties is more sensitive than the sodium fluorescein for detection of capillary MAs. It was even reported that some of the lesions detected by ICGA were very faintly seen or could not be visualized by FA [2, 4, 5]. Moreover, MAs can be seen much better by OCT than FA. The characteristic OCT appearance of MAs is made up of the lumen with variable reflectivity due to different materials surrounded by a round hyperreflective wall. Cystic spaces can be easily distinguished from MAs as they are optically empty [2]. Although OCTA can demonstrate most of the microaneurysms [6], large MAs may not be visualized paradoxically. This limitation is related to the OCTA's sensitivity. When the flow within the lesion is below the slowest value that can be detected by OCTA, it cannot be visualized on OCTA [13]. The decrease in blood flow due to the vessel wall thickening and intraluminal material accumulation has been considered as the cause of poor detection of MAs [4]. OCTA is therefore not a sufficient imaging technique for cases with MAs as in our case.

Since FA is routinely performed in eyes with DR, many MAs can be easily overlooked. Therefore, the presence of MAs cannot be recognized. MAs may not respond well enough to intravitreal anti- vascular endothelial growth factor treatments. Thermal laser photocoagulation has been reported to be more effective in these patients. ICG-guided laser photocoagulation for capillary MAs has been investigated by Paques et al. [5]. In this retrospective study, nine eyes with chronic macular edema and hard exudates due to DR (4 eyes) and RVO (5 eyes) were included. The capillary MAs were demonstrated with OCT and ICGA and its median size was 410 µm (range, 158-603). While four patients had previously received treatment (intravitreal injections and grid laser) for macular edema, none received any treatment within the last 3 months prior to laser treatment. Capillary MAs were selectively photocoagulated using a 532 nm laser (spot size; 50 µm, duration; 0.2 s and power; 100 mW). Photothrombosis inside the lumen was assessed by OCT. Six months after the treatment, the authors reported a significant improvement in visual acuity (mean log MAR, 0.82 versus 0.58) and reduction in macular edema (mean macular thickness, 528 versus 271). In our case, we planned to administer direct focal thermal laser photocoagulation onto the MA together with an intravitreal dexamethasone implant due to severe macular edema as also suggested in the literature but she unfortunately denied any treatment due to reimbursement issues. 

Perifoveal exudative vascular anomalous complex (PEVAC) is a recently defined entity that should be considered in the differential diagnosis of retinal MAs, which occurs as a unilateral, isolated, perifoveal lesion. Large aneurysmal dilatation, hard exudates, intraretinal hemorrhages and fluid accumulation are the common features of both entities. However, the pathogenesis is different. It is considered that PEVAC is resulted from progressive retinal endothelial cell degeneration. In addition, while telangiectatic capillaries usually occur in retinal vascular diseases such as retinal vein occlusion (RVO) and DR, PEVAC is reported in otherwise healthy subjects [14, 15]. The main limitation of this case presentation was lack of treatment and thereby possible treatment effect on MA and macular edema. However, we still believe that multimodal imaging characteristics of the present case with DR related MA were demonstrated in utmost detail. 

## CONCLUSIONS

Clinicians should look for the presence of MAs in patients with severe and chronic macular edema. In light of the present case and the literature review, we believe that FA, ICGA and OCT may be equally helpful for detection and visualizing the extent of MAs. The diagnostic criteria, treatment strategies and prognosis of DR-related MAs are not well-defined. We believe that the definitive diagnosis criteria, treatment alternatives and its prognosis will be better determined by further studies.
